# Aire Disruption Influences the Medullary Thymic Epithelial Cell Transcriptome and Interaction With Thymocytes

**DOI:** 10.3389/fimmu.2018.00964

**Published:** 2018-05-07

**Authors:** Cesar A. Speck-Hernandez, Amanda F. Assis, Rafaela F. Felicio, Larissa Cotrim-Sousa, Nicole Pezzi, Gabriel S. Lopes, Karina F. Bombonato-Prado, Silvana Giuliatti, Geraldo A. Passos

**Affiliations:** ^1^Graduate Programme in Basic and Applied Immunology, Universidade de São Paulo, São Paulo, Brazil; ^2^Molecular Immunogenetics Group, Genetics, Ribeirão Preto Medical School, Universidade de São Paulo, São Paulo, Brazil; ^3^Graduate Programme in Cellular and Molecular Biology, Ribeirão Preto Medical School, Universidade de São Paulo, São Paulo, Brazil; ^4^Morphology, Physiology and Basic Pathology, School of Dentistry of Ribeirão Preto, Universidade de São Paulo, São Paulo, Brazil; ^5^Genetics, Bioinformatics Group, Ribeirão Preto Medical School, Universidade de São Paulo, São Paulo, Brazil

**Keywords:** *Aire* gene, cell adhesion, transcriptome, medullary thymic epithelial cells, immune tolerance

## Abstract

The function of medullary thymic epithelial cells (mTECs) is associated with thymocyte adhesion, which is crucial for the negative selection of autoreactive thymocytes in the thymus. This process represents the root of central tolerance of self-components and prevents the onset of autoimmune diseases. Since thymic epithelia correspond to an important target of donor T cells during the onset of chronic graft-vs-host-disease, mTEC-thymocyte adhesion may have implications for alloimmunity. The *Aire* and *Fezf2* genes function as transcriptome controllers in mTECs. The central question of this study is whether there is a mutual relationship between mTEC-thymocyte adhesion and the control of the mTEC transcriptome and whether Aire is involved in this process. Here, we show that *in vitro* mTEC-thymocyte adhesion causes transcriptome changes in mTECs and upregulates the transcriptional expression of *Aire* and *Fezf2*, as well as cell adhesion-related genes such as *Cd80* or *Tcf7*, among others. Crispr-Cas9-mediated *Aire* gene disruption demonstrated that this gene plays a role in the process of mTEC-thymocyte adhesion. Consistent with the nuclear localization signal (NLS) encoded by *Aire* exon 3, which was targeted, we demonstrate that *Aire* KO^−/−^ mTECs impair AIRE protein localization in the nucleus. Consequently, the loss of function of *Aire* reduced the ability of these cells to adhere to thymocytes. Their transcriptomes differed from their wild-type *Aire*^+/+^ counterparts, even during thymocyte adhesion. A set of mRNA isoforms that encode proteins involved in cell adhesion were also modulated during this process. This demonstrates that both thymocyte interactions and *Aire* influence transcriptome profiling of mTEC cells.

## Introduction

Thymic crosstalk is an active process that involves both cell migration and cell–cell adhesion, during which thymocytes interact with thymic epithelial cells (TECs) and receive signals to proceed with their differentiation (1–3). Because the T cell receptor (TCR) is expressed on the surface of early thymocytes that are located in the thymic cortex and successfully express the TCRβ chain, these cells pass the β-selection checkpoint and then rearrange and express the TCRα chain. Subsequently, double-positive (CD4^+^CD8^+^) cells, which receive weak TCR signals, receive survival signals and undergo positive selection (PS), subsequently becoming single-positive (SP CD4^+^ or CD8^+^) cells. Cortical TECs (cTECs) are in control of the PS of thymocytes (4). The SP cells then migrate to the thymic medulla, and clones expressing self-reactive TCRα/β are eliminated by apoptosis through negative selection (NS), which is closely associated with medullary TECs (mTECs) (4–7). This process involves a specific thymic microenvironment that supports the different stages of T cell development (8). This sequence of events can be traced by using molecular markers, such as for the timing of gene recombination and expression of TCRα/β.

The interaction between TECs and thymocytes, in addition to causing the development and selection of T cells, provides distinct sets of signals that modulate transcriptional gene expression in the different regions of the thymic stroma through which the thymocytes migrate (9). In this context, it is quite appropriate to consider that mTECs represent a unique cell type, as they express an enormous variety of genes and mobilize most of their functional genome (10, 11). The significance of this wide-ranging gene expression is immunological, i.e., it results in self-representation in the thymus though promiscuous gene expression (PGE) (7, 12–15).

The autoimmune regulator (*Aire*) gene is one of the controllers of PGE in mTECs, modulating the so-called *Aire*-dependent genes. Most of these are genes that encode the self-peripheral tissue antigens (PTAs) and are regulated through a mechanism that releases stalled RNA Pol II in the chromatin (16). As RNA Pol II is nonspecific regarding its target mRNAs, this could explain the wide range of mRNAs that *Aire* controls. Moreover, it has been shown that the number of mRNA isoforms that is expressed in mTEC cells is higher than in other cell types and that *Aire* exerts control in the splicing diversity in these cells (17). The implication of these findings concerns the exposure of developing thymocytes to an increased diversity of PTA splice isoforms and diversity of HLA (MHC)-mediated PTA presentation, enforcing NS against self-antigens in the thymus (17–19). Recently, we showed that in addition to PTAs, *Aire* controls the expression of mRNAs that encode cell adhesion proteins in mTEC cells and, consequently, their thymocyte adhesion (20).

A second PGE controller has been identified in mTEC cells, Forebrain embryonic zinc-finger 2 (*Fezf2*), whose encoded protein acts directly on chromatin to modulate the expression of a set of *Aire*-independent genes (21, 22). The sum total of the *Aire*-dependent and the *Aire*-independent genes that are expressed by mTECs is approximately 15,000 genes, representing approximately 62% of the murine functional genome (19). This shows that mTECs are an unusual cell type due to the range of their transcriptome modulation while still maintaining their morpho-functional characteristics. The mTECs are usually identified by flow cytometry and characterized as CD45^−^EpCAM^+^ (epithelial cell adhesion molecule) Ly51^−^, in addition to expressing the cell surface markers MHC II and CD80 (4, 23).

The results obtained with reaggregation thymus organ culture, which is consistent with thymic embryogenesis, show that MHC^−^CD80^−^AIRE^−^ immature mTECs precede MHC^+^CD80^+^AIRE^+^ mature mTEC development (23–25). This indicates that immature cells proceed to mature mTECs though an intermediate stage of *Aire*^−^ cells that begin to express MHC II and CD80, a process that is dependent on RANK-mediated signals from CD4^+^3^−^RANKL^+^ lymphoid cells (4, 23, 26). AIRE^+^ mTECs are short-lived cells; they are post-mitotic and represent the last stage of mTEC differentiation (4, 23, 26). *Aire* ultimately induces apoptosis in mature mTECs, contributing to the diffusion of PTAs within the medullary thymic compartment (23, 26).

Accordingly, mTECs represent an unusual cell type because they express most of their functional genome in the late phase of their differentiation without losing their morpho-functional characteristics (7).

However, it currently remains unclear whether the physical contact with thymocytes influences the broad transcriptional gene expression modulation in mTECs and what the specific participation of *Aire* is in this process.

## Materials and Methods

### Mice and Separation of Thymocytes

We used 4- to 5-week-old female C57BL/6 mice weighing 18–22 g for the surgical removal of the thymus and further thymocyte preparation, which were isolated according to a previously described protocol and whose procedure yielded a thymocyte population with a purity of ≥93% as determined by flow cytometry with a phycoerythrin-labeled anti-CD3 antibody (20, 27, 28). These cells were used for further cell adhesion assays. All experimental procedures followed ethical guidelines under strict guidance and approval from the University of São Paulo Ethics Committee for Animal Experimental Research (Approval # 006/2016-1).

### mTEC Line

We used the *Aire* wild-type (WT) mTEC line (EpCAM^+^, Ly51^−^, UEA-1^+^) mTEC 3.10 cells as previously described (20, 29, 30).

### mTEC-Thymocyte Cell Adhesion Assay

The WT mTEC 3.10 cell line as well as the mutant mTEC 3.10E6 was used in an mTEC-thymocyte adhesion protocol as previously established (31–35) with several modifications introduced by our group (20). Experiments were performed at least six times (six co-cultures with WT mTEC 3.10 cells and six co-cultures with mutant mTEC 3.10E6) with similar results. Then the adhesion index (AI) was calculated as follows: AI = number of adhered thymocytes/number of mTEC cells. Statistical analysis was performed by two-sided Mann–Whitney test with 95% interval.

### Crispr-Cas9-Mediated Aire Indels

#### Crispr-Cas9 Vector, gRNA, and Electrotransfection of mTEC Cells

An all-in-one Crispr-Cas9 vector encompassing gRNA-Cas9-green fluorescent protein (GFP) was designed and purchased from Sigma Aldrich (St. Louis, MO, USA). This vector encodes a specific gRNA, whose complementary sequence is CCCCTTGCTGGTCCCAAGGCCG that targets the *Aire* exon 3 on *Mus musculus* chromosomal location GRCm38:10:78030995–78031017, the Cas9 enzyme, and GFP. The gRNA targeted the CGG PAM motif located immediately upstream of the region within the *Aire* exon 3 that transcribes mRNA nt 346–348 and amino acid residues 113–124 of the AIRE protein. The vector could, in principle, interact with potential off-target gene sequences. Considering the calculated risk for these sequences, which derives from the algorithm proposed by Hsu et al. (36), the gRNA used in this work presents a low off-target probability (Hsu score = 0.7).

Approximately 5 × 10^5^ WT mTEC 3.10 cells were suspended in 82 µl Amaxa Basic Nucleofector™ reagent for primary mammalian epithelial cells (Lonza, Basel, Switzerland) plus 18 µl Lonza supplement containing 5 µg Crispr-Cas9 vector. This cell suspension was placed in an electroporation cuvette and subjected to a single continuous current electric pulse of 200 V during 30 ms in a BTX Square Electroporator (Holliston, MA, USA). Electrotransfected cells were seeded in a well of a six-well Costar^®^ culture plate (Corning) containing 3 ml RPMI medium supplemented with 10% inactivated fetal bovine serum (FBS) plus antibiotics and cultured for 24 h in a 37°C incubator with a 5% CO_2_ atmosphere and then trypsinized as mentioned above. Wild-type mTEC 3.10 cells electroporated only in the presence of the Nucleofector^®^ reagent plus Lonza supplement were cultured as above and were considered control cells.

The GFP^+^ cells were separated though a FACS Aria III flow cytometer (Becton Dickinson, Franklin Lakes, NJ, USA). Individual cells were automatically and consecutively placed in separate wells of a polystyrene flat-bottomed 96-well plate (Corning) containing 100 µl RPMI medium supplemented with 10% FBS plus antibiotics and cultured. Data were analyzed *via* Beckman Coulter Kaluza software.^1^ Cell proliferation was observed though a conventional inverted microscope during a period of approximately 3 weeks, exchanging fresh culture medium every other day.

The surviving clones from the complete process, i.e., electroporation, sorting, and single cell culture were numbered, cultured until confluence and transferred to 24-well plate, then to 6-well plate, and finally to 25 cm^2^ culture bottles. Confluent cells were removed from culture bottles by trypsin treatment, processed by a conventional cryopreservation protocol that includes dimethyl sulfoxide, and stocked in liquid N_2_ for further analysis.

#### Identification and Characterization of Aire Exon 3 Mutant Clones

The genomic DNA (200 µg) from individual GFP^+^ mTEC clones was used in conventional PCR for the amplification of a 415 bp genomic DNA fragment encompassing the *Aire* exon 3 (Ensembl acc ENSMUSG00000000731), whose primer (forward F or reverse R) sequences were: F = 5′ CCAATGGGTAGCATCGG 3′ and R = 5′ CTCTTGAGTGTACCTGGGCTG 3′. The Primer3 web tool^2^ was used to select pairs of oligonucleotide primers with an optimal melting temperature of 60°C.

A 50 µl PCR mixture containing the input genomic DNA, PCR buffer, primers, dNTPs, and Taq enzyme (Gotaq G2 Flexi DNA Polymerase, Promega) was subjected to the following thermal cycling conditions: 30× (95°C 20 s, 54°C 30 s, 72°C 30 s), 1× (72°C 5 min). The PCR reactions were performed in triplicate for each GFP^+^ clone. Genomic DNA from untransfected mTEC 3.10 cells was used as a negative control.

The PCR products were purified using a QIAquick PCR Purification Kit (Qiagen, Hilden, Germany). For the identification of mutant clones, the respective PCR products were digested with T7 endonuclease (New England Biolabs, Ipswich, MA, USA) following the manufacturer’s instructions. The digestion fragments were resolved though microfluidic electrophoresis using Agilent DNA nanochips and an Agilent 2100 bioanalyzer (Agilent Technologies, Santa Clara, CA, USA). As the T7 endonuclease cleaves mismatched double-stranded PCR products, the DNA of mutant clones were identified by the presence of two bands of approx. 200 bp cut PCR product, in contrast with WT DNA, which featured just one band of undigested DNA.

The PCR products of *Aire* mutant clones were processed by Sanger sequencing for further characterization of mutations. Sanger electropherograms were analyzed using the Crisp-ID program^3^ (37) for exact indel size and location in the Crispr-Cas9 *Aire* exon 3-targeted region.

FASTA sequences of a mutant clone selected for further analysis in this work, here termed mTEC 3.10E6, were deposited at GenBank^4^ under accession numbers (acc MG493266 for the *Aire* mutant allele 1 and acc MG493265 for the *Aire* mutant allele 2).

#### Translation of the DNA Sequences Into Protein Sequences

We initially used the Uniprot databank^5^ to recover the primary WT sequence of the AIRE protein under acc Q9Z0E3. Then, the FASTA DNA sequences of the mutant 3.10E6 (allele 1 and allele 2) were translated into proteins by using the Expasy Translate Tool.^6^

To predict the effect of mutations on the amino acid sequence of the encoded AIRE protein, we used the Provean tool,^7^ which is a software often used to characterize the functional effects of amino acid variations (substitutions or deletions) on proteins. AIRE primary protein sequences (WT and mutant) were aligned and compared by using the Clustal Omega program.^8^

### Total RNA Preparation

Total RNA of *Aire* WT (mTEC 3.10) or *Aire* mutant (mTEC 3.10E6) cells was prepared using the mirVana kit^®^ (Ambion, Austin, TX, USA) according to the manufacturer’s instructions. Evaluation of RNA integrity was performed by microfluidic electrophoresis using Agilent RNA 6000 nano chips and an Agilent 2100 bioanalyzer (Agilent Technologies Santa Clara, CA, USA) as previously described by our group (20). Only RNA samples that were free of proteins and phenol and had an RNA Integrity Number ≥9.0 were selected for cDNA synthesis using SuperScript reverse transcriptase enzyme according to the manufacturer’s instructions (Invitrogen Corporation, Carlsbad, CA, USA).

### Reverse Transcription Quantitative Real-Time PCR (RT-qPCR)

The confirmation of transcriptional expression of focused genes was assayed by RT-qPCR. The expression level of each target gene was normalized to the housekeeping gene *Hprt*, which is commonly used as a reference. The Primer3 web tool (see text footnote 2) was used to select pairs of oligonucleotide primers spanning an intron/exon junction with an optimal melting temperature of 60°C.

The respective sequences were retrieved from the NCBI GenBank database.^9^ The forward (F) and reverse (R) primer sequences (presented in the 5′–3′ orientation) were the same as those previously used in our group (20) as follows: *Aire* (acc NM_001271549.1) F = GAAGCTGTACCCACCTCTGG, R = ATTGAGGAGGGACTCCAGGT, *Fezf2* (acc NM_NM_080433.3NM) F = GAACGAGGGGGAGTCAAGAG, R = TCTAGCTCCGGTGTGGACAG, *CD80* (acc NM_009855.2) F = CCTGGGAAAAACCCCCAGAA, R = ACAACGATGACGACGACTGT, *Nfkbia* (acc NM_010907.2) F = AGGACGAGGAGTACGAGCAA, R = CGTGGATGATTGCCAAGTGC, and *Tcf7* (acc NM_001313981.1) F = CTGTCCCCTTCCTGCGGATA, R = GTCCAGGTACACCAGATCCC.

Gene expression was quantified using a StepOne Real-Time PCR System (Applied Biosystems, USA). The analyses were performed using the cycle threshold (*C*_t_) method, which allows for quantitative analysis of the expression of a factor using the formula 2^−ΔΔCt^, in which Δ*C*_t_ = *C*_t_ target gene − *C*_t_ of the housekeeping gene *Hprt*, and ΔΔ*C*_t_ = Δ*C*_t_ sample − Δ*C*_t_. Experiments were performed in six independent replicates and statistical analysis of the data was made through the Mann–Whitney two-sided test with 95% interval.

### Western Blotting of AIRE Protein

Western blotting analysis of AIRE protein levels was performed according to a conventional protocol, which was described in a previous work from our group (20), including electrotransfer of the SDS-PAGE of total proteins extracted from *Aire* WT (mTEC 3.10) or *Aire* mutant (mTEC 3.10E6) to a polyvinylidene fluoride membrane (BioRad, Hercules, CA, USA), incubation with anti-AIRE-1 primary antibody (C-2 mouse monoclonal IgG_1_ kappa light chain, Santa Cruz Biotechnology, Dallas, TX, USA) and developing for AIRE protein band visualization.

### Immunolocalization of AIRE Protein in mTECs

Immunolocalization of AIRE protein in *Aire* WT (mTEC 3.10) or *Aire* mutant (mTEC 3.10E6) cells was performed according to a conventional immunofluorescence protocol, which was described in previous work from our group (20) using a goat anti-mouse AIRE D17 IgG polyclonal primary antibody (Santa Cruz Biotechnology) and Novex™ mouse anti-goat IgG rhodamine red conjugate (Life Technologies Corp., Carlsbad, CA, USA) as a secondary antibody. To visualize the cytoplasmic region, actin filaments were labeled with AlexaFluor^®^ 488-conjugated phaloidin (Life Technologies) according to the manufacturer’s instructions. The nuclei were labeled with DAPI. In this study, cells were observed using an Apo Tome immunofluorescence microscope (Zeiss, Oberkochen, Germany). We counted 15 microscopic fields totalizing approximately 150 each cell type.

### Statistical Analysis of the Data

Quantitative results of RT-qPCR and adhesion assay (from independent experiments performed six times) were analyzed by Mann–Whitney test with 95% confidence intervals. The IBM SPSS Statistics program^10^ was used for calculations and graphing of results.

### Transcriptome Analysis Through RNA-Seq

We followed a as protocol previously described (38). Briefly, paired-end (2 × 100 bp) sequencing was performed using an Illumina HiSeq 2500 sequencer (Illumina, San Diego, CA, USA) using a TruSeq Stranded Total RNA Library Prep Kit (Illumina).

The quality of raw FASTQ sequences was first analyzed through a FASTQC program.^11^ Then, FASTQ sequences were mapped to the *Mus musculus* reference genome (mm10) using the STAR 2.5 Spliced Aligner program,^12^ which output a BAM file containing the sequences and their genomic references and a GTF file with gene annotations used for further determinations of the number of reads per transcript through the HTSeq Count program.^13^

For each RNA sample analyzed, we recovered a list of genes and their respective number of transcripts that served as input for determinations of the differentially expressed (DE) mRNAs through the DESEq2 package^14^ within the R platform.^15^ DESEq2 calculates the fold change (FC) for each mRNA considering a contrast matrix for a given experimental condition. In this study, we defined adhesion as a contrast variable and DE as those mRNAs with a *p* value <0.05 and false discovery rate (FDR Benjamini–Hochberg correction) and FC ≥1.5.

The DE mRNAs were hierarchically clustered, and a heat-map was constructed to evaluate the expression profiling. The Euclidean distance and the complete linkage method were used for clustering the samples and mRNAs using the R platform.

We used the RSEM software^16^ to estimate isoform abundance from the mapped reads as generated from the STAR 2.5 Spliced Aligner program as described above. The STAR output Aligned.to.Transcriptome.out.bam files were used as RSEM input to generate isoform counts for each RNA sample analyzed. Then, we compared isoform expression using the EbSeq package^17^ within the R platform. DE isoforms were determined by comparing each two conditions considering as significant an adjusted FDR *p* value < 0.05 and an FC ≥ 1.5. The RNA-sequencing data of this work are available on Gene Expression Omnibus^18^ under the accession number GSE91015.

### Functional Enrichment of DE mRNAs

The list of the DE mRNAs was analyzed in terms of functional enrichment through the Database for Annotation, Visualization, and Integrated Discovery (DAVID) annotation tool.^19^ This tool was used for the identification of the main biological processes and pathways represented by DE mRNAs. A functional category was considered significant if it had at least three mRNAs and a score of *p* < 0.005 with Benjamini–Hochberg correction.

## Results

### Sorting of GFP^+^ Crispr-Cas9-Transfected mTEC Cells and T7 Endonuclease Screening

The electroporation and nucleofection process with a vector simultaneously expressing GFP, Casp9, and a gRNA targeting *Aire* exon 3 allowed a satisfactory number of GFP^+^ cells to be isolated, in light of the fact that the main objective of this procedure was to obtain at least one mTEC mutant clone. Only mTEC cells transfected with the Crispr-Cas9 vector expressed GFP, which allowed their isolation by flow cytometry (Figure S1 in Supplementary Material). Of the 87 single cells deposited in a 96-well plate, we recovered nine clones for further evaluation of the occurrence of mutations in *Aire* exon 3. We then amplified a 415 bp PCR product that encompassed *Aire* exon 3, which was then digested with the enzyme T7 endonuclease. Of these nine clones, we were able to identify two that had their PCR DNA product digested, indicating the occurrence of mutations; one clone was named “3.10E6,” which was selected for this study (Figure S2 in Supplementary Material).

### Sanger Sequencing and Characterization of Crispr-Cas9-Induced Mutations

For this study, we selected clone 3.10E6 for further characterization through Sanger sequencing of the *Aire* exon 3-targeted region. Figure S3 in Supplementary Material shows the respective sequencing electropherograms of mTEC CT1 cells (control untransfected WT mTEC 3.10 cells), 3.10A6 (a Crispr-Cas9 vector-transfected GFP^+^ clone but whose PCR product of the *Aire* exon 3 was resistant to the T7 endonuclease), and 3.10E6 (a Crispr-Cas9 vector-transfected GFP^+^ clone whose PCR product of the *Aire* exon 3 was digested by the T7 endonuclease).

**Figure 1:**
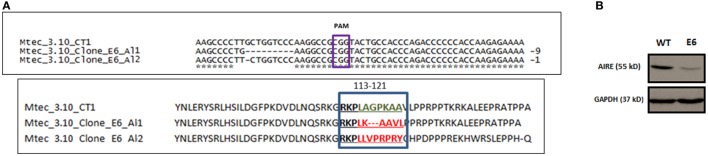
DNA sequence alignment of *Mus musculus Aire* exon 3 CRISPR-Cas9 target region nearing PAM CGG sequence for wild-type (WT) medullary thymic epithelial cell (mTEC) 3.10 and mTEC 3.10E6 for the characterization of indel size and location (allele 1 and allele 2) and AIRE protein translation from the respective DNA segments **(A)**. Western blot (WB) of SDS-PAGE WT mTEC 3.10 and mTEC 3.10E6 mutant clone cell lysates for detection of *Mus musculus* AIRE protein expression. WB membrane was probed with an antibody against AIRE protein (upper panel), washed, and then probed with an antibody against GAPDH that was used as an internal load control. This procedure allowed to show that the mTEC 3.10E6 mutant clone express relatively less AIRE protein than its WT mTEC 3.10 counterpart **(B)**.

CRISP-ID program analysis of the Sanger electropherograms showed that no mutations were found in the WT mTEC 3.10 CT1 or clone 3.10A6, whereas clone 3.10E6 was characterized as a carrier of indel mutations affecting both *Aire* alleles [knockout (KO) compound heterozygosis]. This clone was subsequently selected for further analysis. In *Aire* allele 1, there were two types of mutations: T>G substitution (mRNA position 351) followed by a nine-bp deletion (GCTGGTCCC, mRNA positions 352–360) that transcribed a 1,647 nt *Aire* mRNA; in allele 2, there was a single G deletion at mRNA position 352 that transcribed a 1,655 nt *Aire* mRNA (Figure 1A).

*In silico* AIRE protein translation and sequence alignment through the Clustal Omega program (Figure S4 in Supplementary Material) showed that T>G nucleotide substitution in allele 1 resulted in a L118L silent mutation, whereas the nine bp deletion provoked a frameshift leading to deletion of three amino acid residues (A119_P121del) and a consequently shorter 548 amino acid (aa) residue AIRE protein (Figure 1A).

Analysis using the Provean program, which characterizes the functional effects of protein variations from amino acid substitutions or deletions based on calculations of sequence alignments, considers that protein variants featuring a Provean score ≤−2.5 are deleterious. The T>G substitution in allele 1 resulted in no alteration (L118L) in the amino acid sequence (Provean score: −1.46), whereas the deletion of the three amino acid residues (A119_P121del) was deleterious for the AIRE protein nuclear localization signal (NLS) (Provean score: −10.02).

Regarding allele 2, the G nucleotide deletion resulted in a TGA stop codon at mRNA position 352 and, consequently, a 158-aa truncated AIRE protein. For this reason, Provean analysis for allele 2 could not be performed (Table S1 in Supplementary Material).

Moreover, as the anti-AIRE primary antibody used in Western blot (WB) analysis was raised against amino acids 246–545 mapping the C-terminus of AIRE, it could not recognize the truncated AIRE protein from allele 2. The WB depicted in Figure 1B shows the expression of WT AIRE protein in mTEC 3.10 cells and the mutant AIRE in mTEC 3.10E6 clone. Figure S5 in Supplementary Material shows the full WB membrane image.

According to these analyses, clone mTEC 3.10E6 can be considered an *Aire* compound heterozygous KO that expresses a deleterious AIRE protein from the allele 1. Accordingly, this clone was selected for further study.

### Crispr-Cas9-Mediated Mutations Disturb Aire mRNA Expression, Protein Expression, and Nuclear Localization

**Figure 2:**
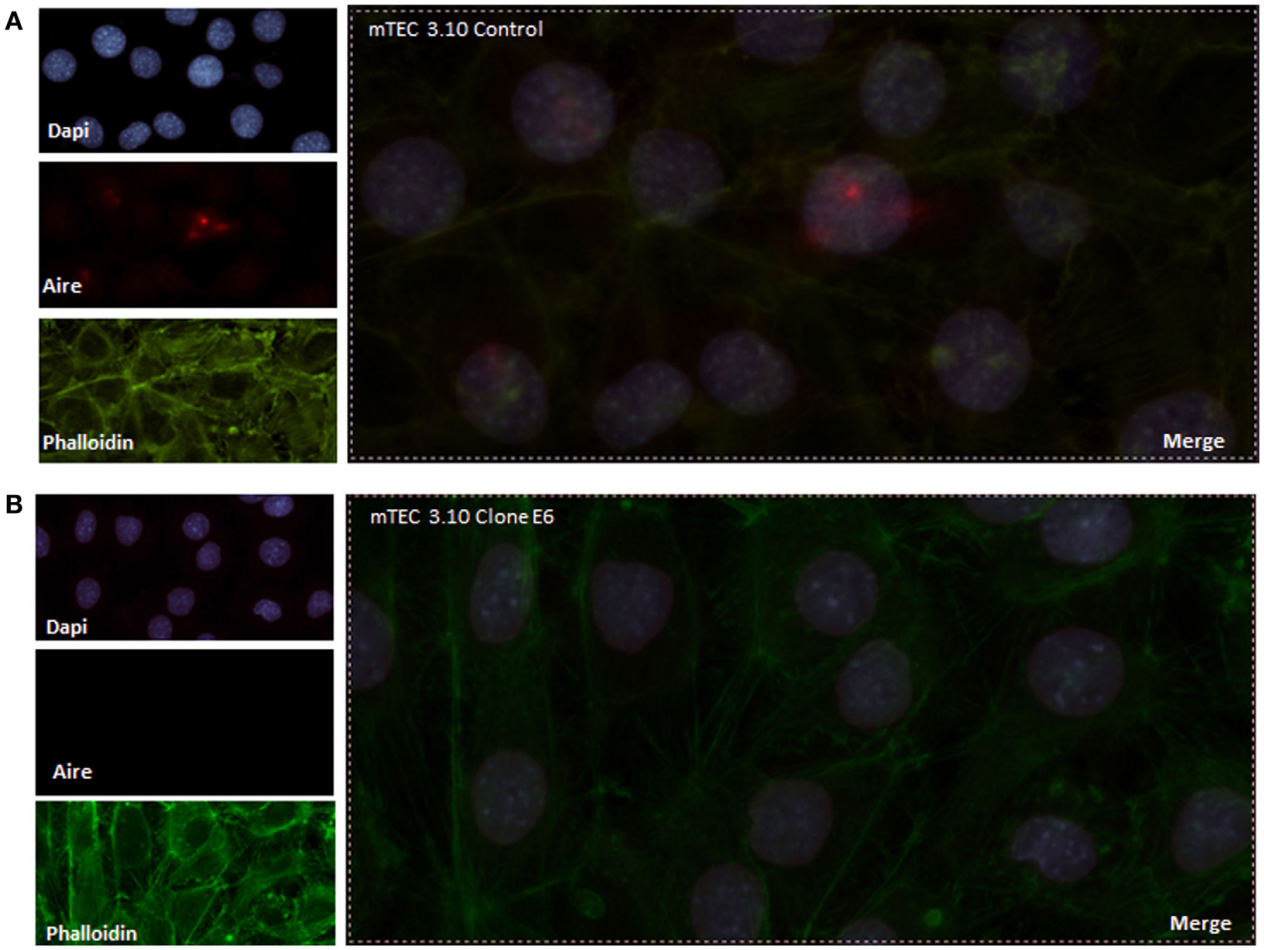
Immunofluorescence of AIRE protein showed that wild-type medullary thymic epithelial cell (mTEC) 3.10 cells exhibited AIRE protein nuclear localization **(A)** while mTEC 3.10E6 mutant clone did not showed nuclear staining for this protein **(B)**. Cells were labeled with an antibody against *Mus musculus* AIRE protein (red dots), with phalloidin to show the cytoplasmic region and with DAPI to show nuclei (blue).

**Figure 3:**
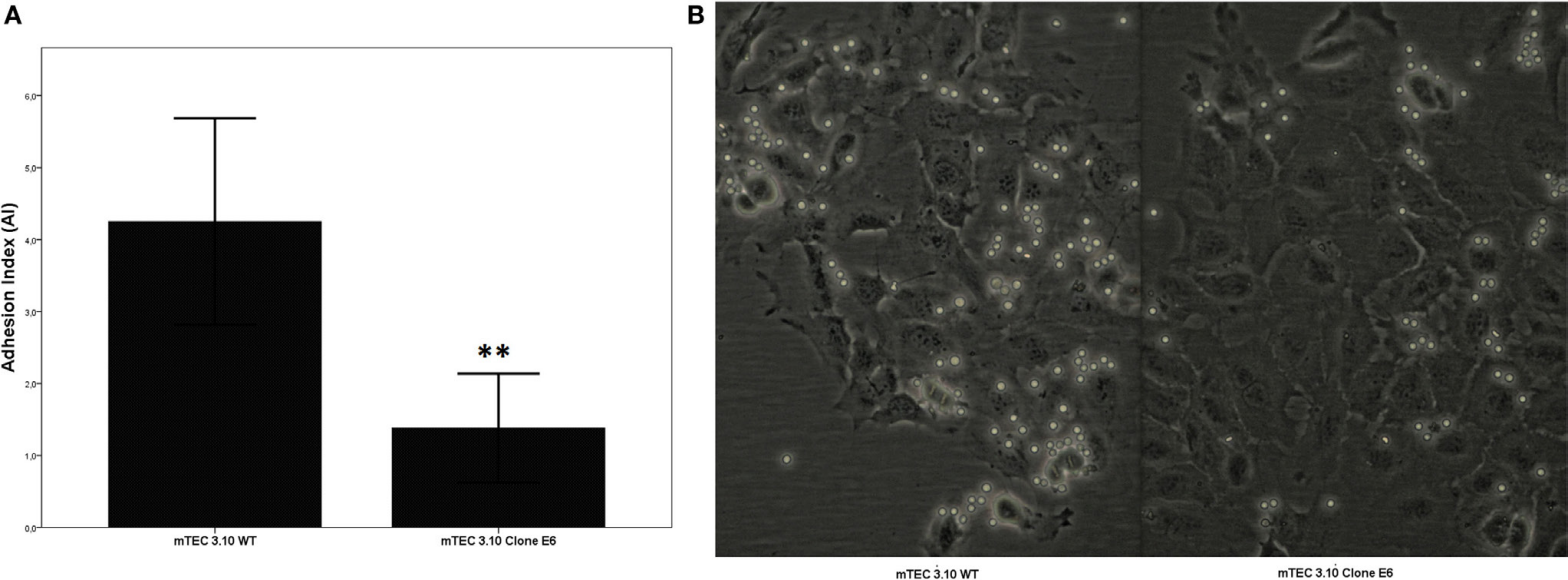
The medullary thymic epithelial cell (mTEC)-thymocyte adhesion assay showed decrease of thymocyte interaction with mTEC 3.10E6 mutant clone in comparison with its wild-type (WT) mTEC 3.10 counterpart. The adhesion degree is plotted as adhesion index (AI), in which values correspond to mean ± SD from three independent experiments and were significantly different between control (WT) vs mutant cells. Mann–Whitney two-sided test with 95% interval **(A)**. Photomicrography of fresh mTEC-thymocyte co-cultures comparing WT mTEC 3.10 vs mutant mTEC 3.10E6 **(B)**.

To evaluate the extension of the effects of Crispr-Cas9-mediated mutations, we evaluated the relative levels of *Aire* mRNA transcripts by RT-qPCR and AIRE protein expression by WB analysis. Our results showed that clone 3.10E6 expresses significantly less AIRE protein than its mTEC 3.10 cell line WT counterpart (Figure 1B). Immunolocalization was used to assess whether such a reduction would affect the protein nuclear localization. Compared to the WT mTEC 3.10 cell line, which featured specked red granules of AIRE protein associated with chromatin (50 AIRE^+^ cells/150 analyzed cells) in immunofluorescence images, the 3.10E6 cells did not exhibit any AIRE protein granules in the nucleus (zero AIRE^+^ cells/150 analyzed cells) (Figures 2 and 3A).

### Mutant Clone 3.10E6 Cells Have Decreased Thymocyte Adhesion

Medullary thymic epithelial cell (mTEC)-thymocyte cell adhesion was used to assess the functional consequences of Crispr-Cas9-mediated *Aire* mutation. Figure 3 shows that clone 3.10E6 had a significantly reduced ability to adhere to thymocytes.

### mTEC-Thymocyte Adhesion Influences Transcriptional Expression of Aire, Fezf2, and Cell Adhesion Genes

**Figure 4:**
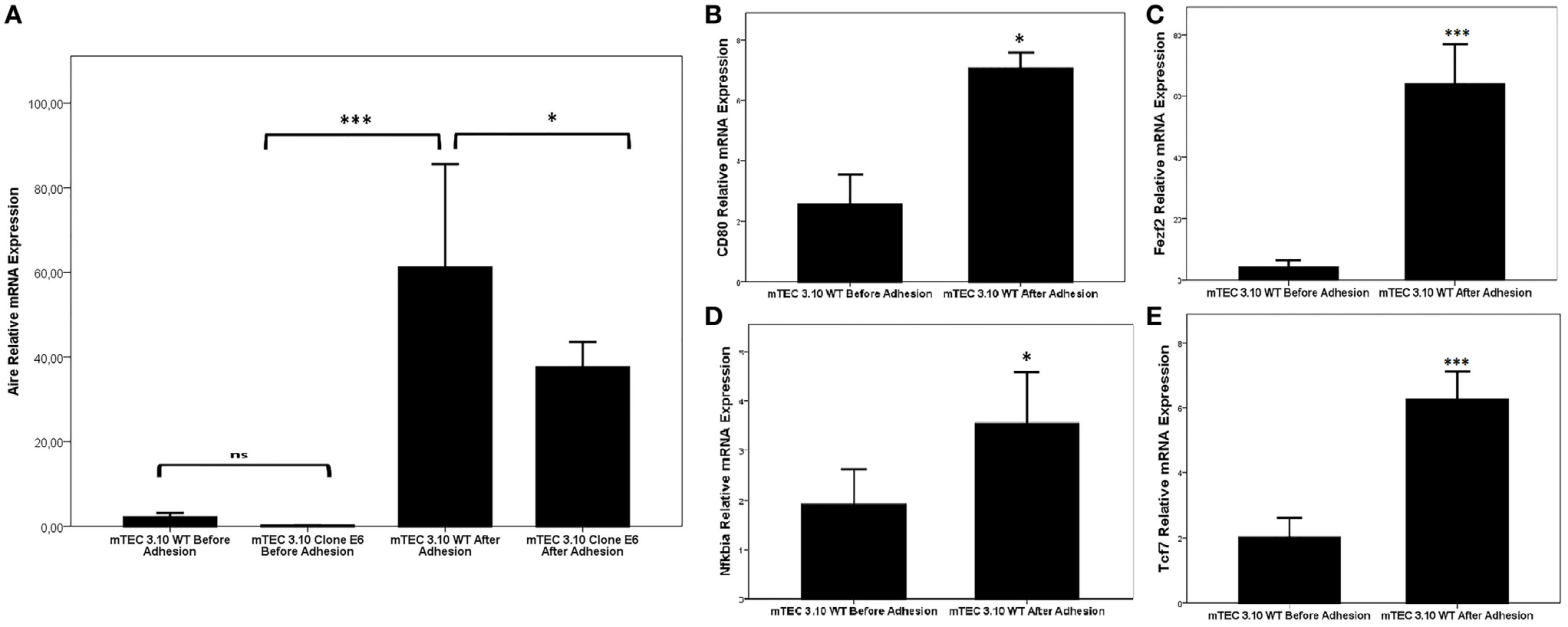
Relative expression of *Aire* **(A)**, *Fezf2* **(B)**, *Cd*80 **(C)**, *Nfkbia* **(D)**, and *Tcf7* **(E)** mRNAs in wild-type 3.10 or mutant 3.10E6 medullary thymic epithelial cells (mTECs) before and after thymocyte adhesion. The respective transcriptional expression of these mRNAs were quantified by real-time quantitative-PCR (RT-qPCR). Data were normalized using Hprt mRNA levels and are presented as mean ± SD from six independent experiments. Mann–Whitney two-sided test with 95% interval.

Figure 4 shows that the WT mTEC 3.10 cell line expresses reduced levels of *Aire* mRNA transcripts, but comparatively and significantly more than clone 3.10E6. When mTEC 3.10 cells adhere to thymocytes, the transcriptional expression of *Aire* increases significantly. Similar modulation of *Aire* expression was observed when clone 3.10E6 cells adhered to thymocytes. However, when comparing the levels of *Aire* expression between mTEC 3.10 cells and clone 3.10E6 after thymocyte adhesion, we observed that the mutant clone showed reduced *Aire* expression.

We then evaluated whether thymocyte adhesion itself, independent of *Aire*, influences specific transcriptional expression in the WT mTEC 3.10 cell line. Figure 4 shows that *Aire* (Figure 4A), *Cd80* (Figure 4B), *Fezf2* (Figure 4C), *Nfkbia* (Figure 4D), and *Tcf7* (Figure 4E) were significantly upregulated in mTECs after their adhesion with thymocytes.

### AIRE Protein Nuclear Localization Is Increased With mTEC-Thymocyte Adhesion

**Figure 5:**
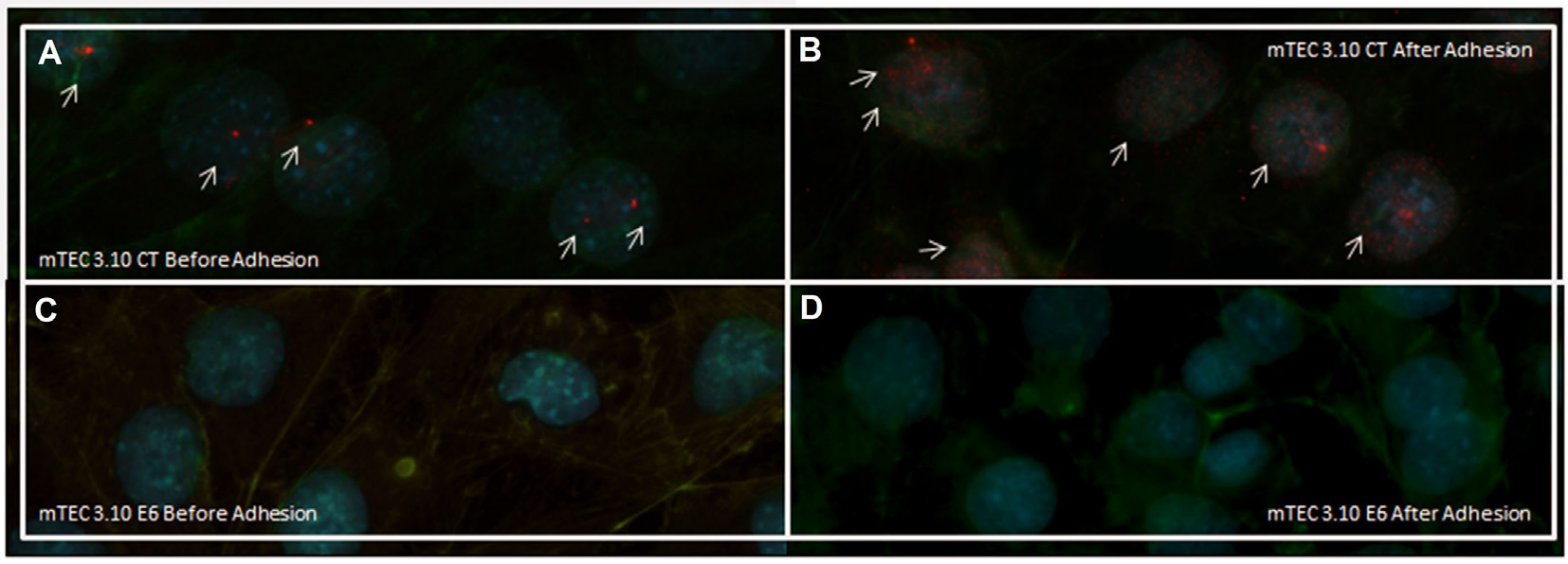
Immunofluorescence of AIRE protein in wild-type (WT) medullary thymic epithelial cell (mTEC) 3.10 cells before and after thymocyte adhesion **(A,B)** or in mTEC 3.10E6 mutant clone **(C,D)**. Thymocyte adhesion increased AIRE protein nuclear localization in WT mTECs while the mTEC 3.10E6 mutant clone did not exhibited nuclear AIRE protein staining neither before of after thymocyte adhesion. Cells were labeled with an antibody against *Mus musculus* AIRE protein (red dots), with phalloidin to show the cytoplasmic region and with DAPI to show nuclei (blue).

After confirming that thymocyte adhesion increases the transcriptional expression of *Aire*, we used immunolocalization to assess whether such an increase would affect the nuclear localization of the AIRE protein. Figures 5A,B shows that the mTEC 3.10 cell line demonstrated increased nuclear localization of the AIRE protein after adhesion with thymocytes, with granules of AIRE protein associated with chromatin (red dots in the immunofluorescence image). However, clone 3.10E6 cells were negative for AIRE protein localization even after adhesion with thymocytes, showing no red dots in the immunofluorescence images (Figures 5C,D).

### The Transcriptome of mTEC Cells Is Dependent on Aire and Thymocyte Adhesion

Comparative analyses were made for various combinations of the study variables [*Aire* WT (before or after thymocyte adhesion) vs *Aire* KO (before or after thymocyte adhesion)]. Initially, we performed comparisons between the WT mTEC 3.10 cell line and the mutant 3.10E6 transcriptome through Euclidean distance heat-maps to evaluate how similar the transcriptomes were between the samples as a whole. Figure S4 in Supplementary Material shows that the transcriptomes of the WT mTEC 3.10 cell line and mutant clone 3.10E6 were significantly different, as the two types of samples were positioned in separate clusters, even considering the conditions before and after adhesion with thymocytes.

**Figure 6:**
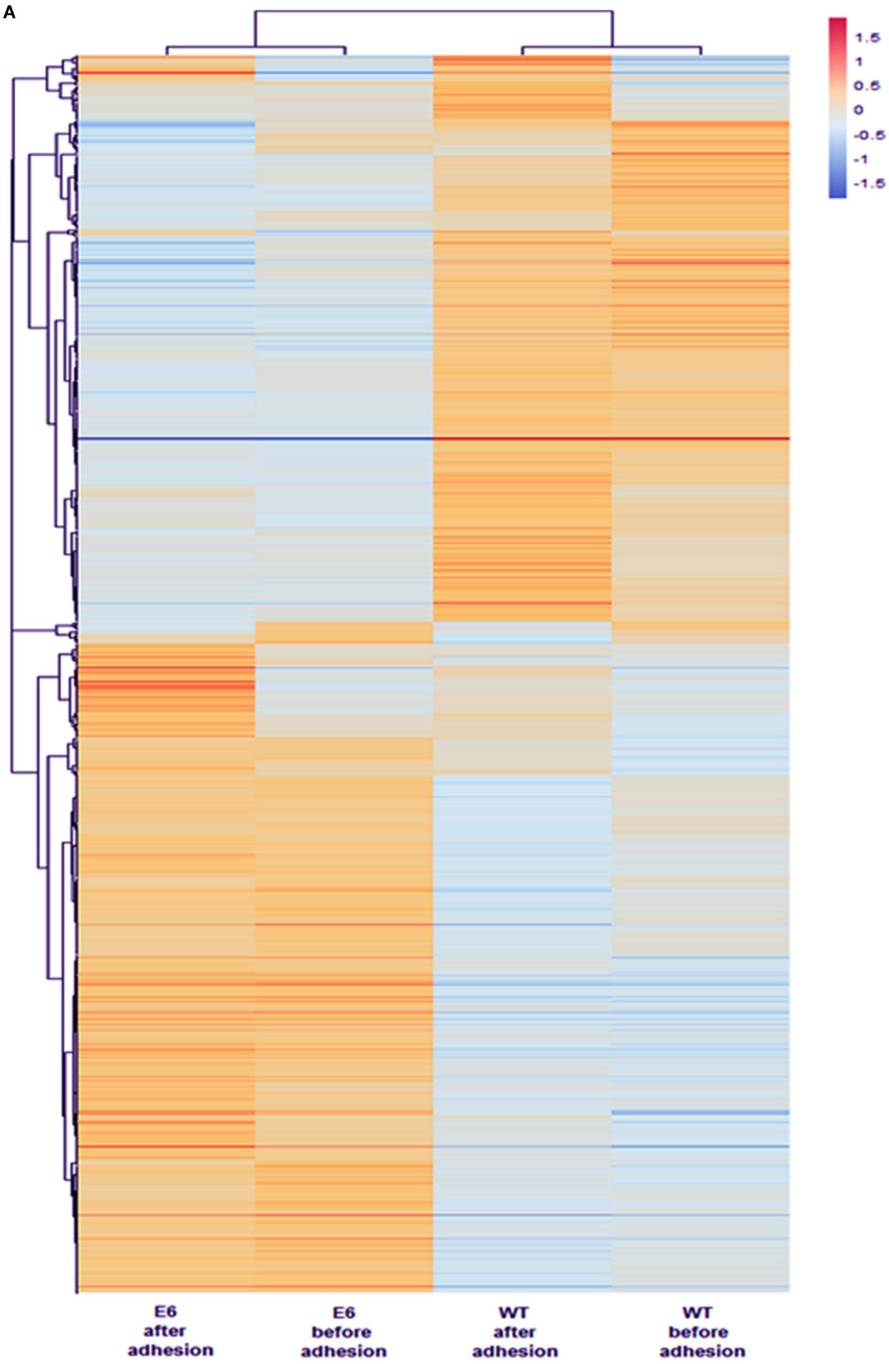

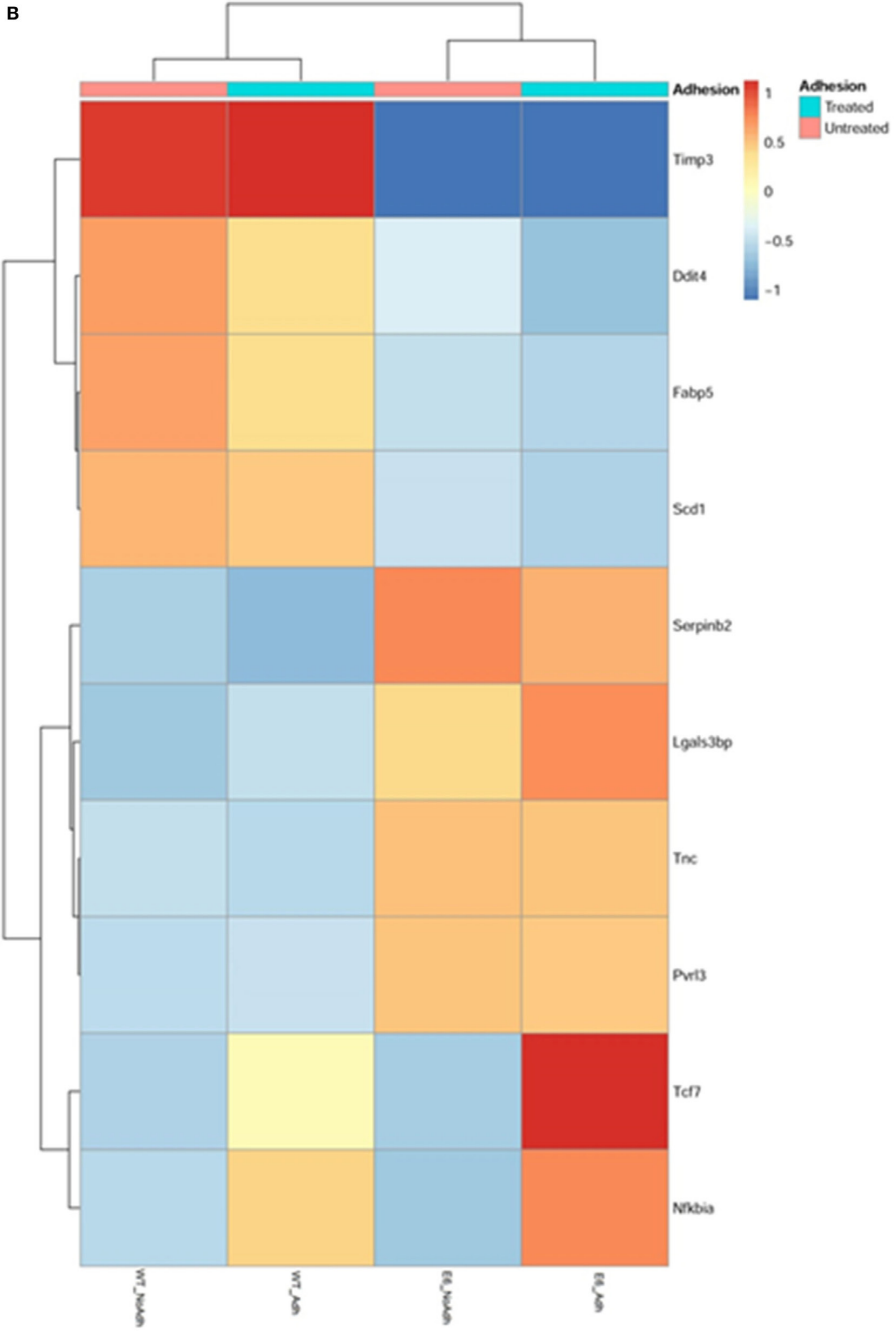

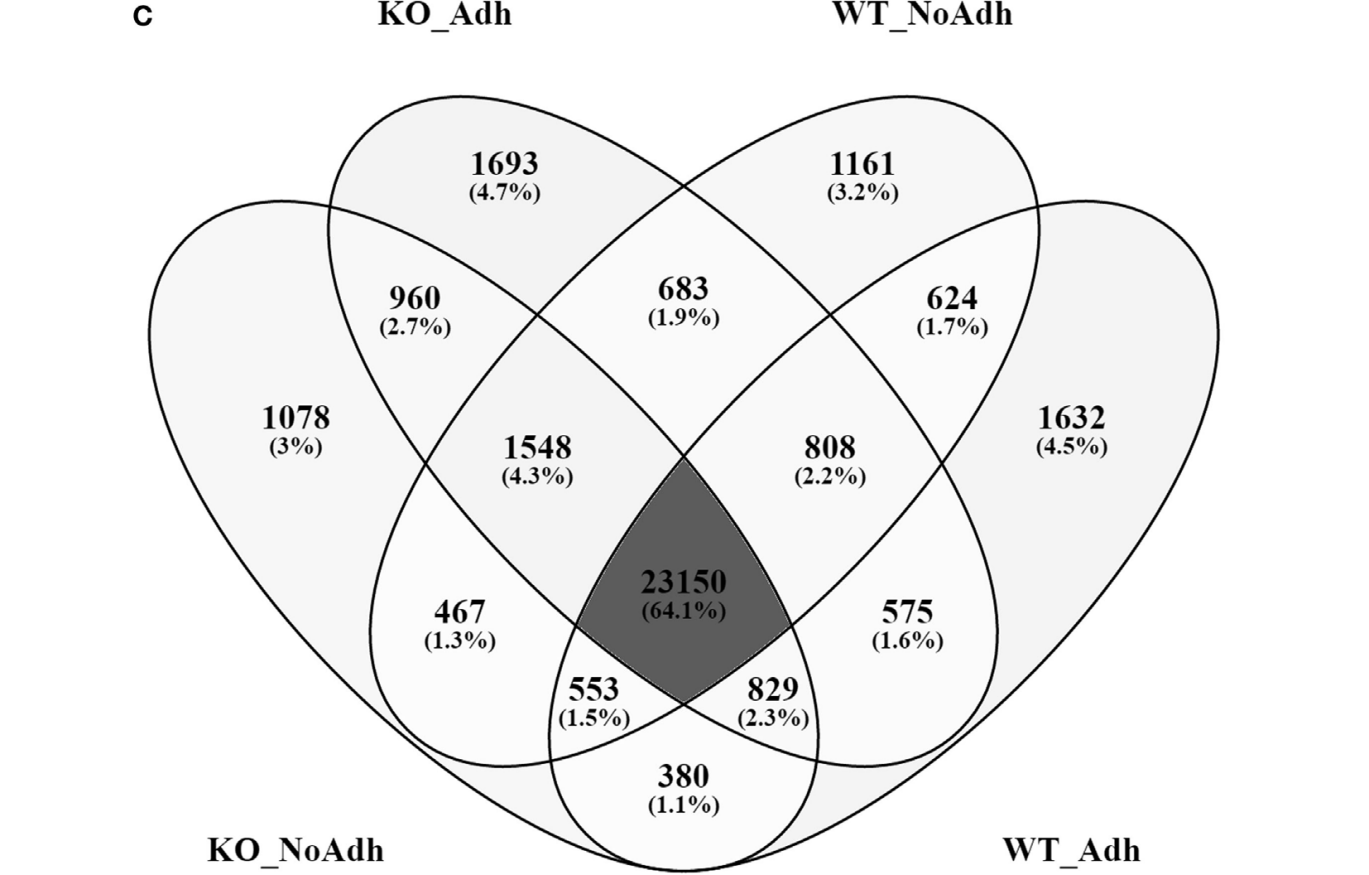
The mRNA transcriptome profile of wild-type (WT) medullary thymic epithelial cell (mTEC) 3.10 or mTEC 3.10E6 mutant clone as evaluated by RNA-Seq. The expression profiling of 902 differentially expressed mRNAs comparing WT mTEC 3.10 cells with mTEC 3.10 mutant clone, before and after thymocyte adhesion **(A)**. The top 10 mRNAs presenting higher expression differences considering the thymocyte adhesion as a variable. Dendrograms and heat-maps were constructed using R platform. Red = upregulation, blue = downregulation [fold change ≥ 1.5 and false discovery rate (FDR) < 0.05] **(B)**. Venn diagram showing the abundance of tissue-specific isoforms when comparing WT mTEC 3.10 cells before (WT_NoAdh) and after (WT_Adh) adhesion and mTEC 3.10E6 mutant clone before (KO_NoAdh) and after (KO_NoAdh) thymocyte adhesion (transcripts per million > 1.0) **(C)**.

Next, the samples and DE mRNAs were hierarchically clustered, and a heat-map was constructed to evaluate the individual expression profiles. Figure 6A shows the expression profiling of 902 DE mRNAs between the WT mTEC 3.10 cell line and mutant clone mTEC 3.10E6. Figure 6B shows the top-10 mRNAs (*Timp3, Ddit4, Fabp5, Scd1, Serpin2, Lgals3bp, Tnc, Pvrl3, Tcf7*, and *Nfkbia*), whose biological functions, identified through the DAVID databank, are associated with cell adhesion, that featured significant differences in their expression levels.

Moreover, we evaluated the effects of *Aire* or thymocyte adhesion on the modulation of mRNA isoforms in mTECs under the different conditions studied. Figure 6C indicates a Venn diagram that shows the number of different isoforms (isoform abundance) and the respective percentages relative to the total isoforms. The different experimental conditions share most of the detected isoforms, but each of the conditions also features populations of specific transcripts.

The different experimental conditions and DE mRNA isoforms were then hierarchically clustered, and heat-maps were constructed to visualize the individual expression profiles. This allowed us to evaluate the influence that *Aire* has on the modulation of mRNA isoforms that encode proteins involved in cell adhesion and, ultimately, the influence that thymocyte adhesion has on the transcriptomes of mTECs. The top-four DE mRNAs that featured greater isoform abundance involved with cell adhesion were *Cd44, Timp3, Tnc*, and *Tcf7*.

## Discussion

In this study, we used mTEC-thymocyte adhesion assays and generated Crispr-Cas9-mediated indel mutations to evaluate the hypothesis that *Aire* and thymocyte interactions function synergistically in the regulation of cell adhesion-related genes and their isoforms and in comprehensive mTEC mRNA transcriptome modulation.

We have previously observed that *Aire* controls the expression of genes involved in cell adhesion in mTECs, and its partial inhibition through a small interfering RNA (anti *Aire* siRNA) system disturbs mTEC-thymocyte adhesion. A reduction in *Aire* in mTECs was sufficient to decrease the expression of genes, such as *Cd80, Vcam, Icam4, Col2a1, Ccl3*, and *Lama1*, impairing adhesion with thymocytes (20). This demonstrated that variations in *Aire* expression affect mTEC-thymocyte adhesion, which is an essential property for the induction of immunological tolerance.

However, two questions remained: (1) do deleterious mutations in *Aire* and (2) adhesion to thymocytes affect the mTEC transcriptome, including cell adhesion genes?

More than 100 mutations throughout the human *Aire* gene sequence, affecting all 14 of its exons, have been described in association with clinical manifestations of autoimmune APECED syndrome (39). Nevertheless, the consequences of *Aire* mutation in mTECs regarding their adhesion to thymocytes are unknown.

The existing *Aire* KO mouse model (*Aire*^−/−^), which involves a deletion of *Aire* exon 2 and results in the production of a truncated protein with loss of the SAND and PHD-1 and PHD-2 domains (40, 41), could hypothetically be used to approach these questions.

However, as freshly isolated mTEC cells from the thymus (primary cells) are not easily maintained in culture, and considering that one of our objectives was to make use of the *in vitro* mTEC-thymocyte adhesion assay, the mTEC cells of these classical *Aire* KO mice would not be adequate for the present study. For these reasons, we used the mTEC 3.10 cell line (CD45^−^, EpCam^+^, Lyn51^−^, UEA1^+^), which has been used in previous studies from our laboratory (20, 42).

We therefore used four main experimental and/or bioinformatics approaches to evaluate the hypothesis of this work: (1) the Crispr-Cas9 system to generate mutations within *Aire* exon 3, (2) the prediction of loss-of-function of the mutant AIRE protein, (3) mTEC-thymocyte adhesion assays, and (4) mRNA transcriptome profiling, including mRNA isoforms.

Interest in *Aire* exon 3 developed because it encodes the NLS domain of the AIRE protein, a domain involved in translocating that protein from the cytoplasm to the nucleus of mTECs (43–46). Mutations in this region affect not only the transport of the protein to the nucleus but also disrupt its transcriptional behavior (47, 48). The location of the NLS domain involves the amino acid residues at positions 110–114 and 131–133 of the AIRE protein (45). Indel-like mutations in this region may result in truncation in the AIRE protein, causing a loss of the adjacent SAND or PHD1–2 domains (14, 49) (Figure 1A).

Analyses performed with Provean software predicted that clone 3.10E6 showed partial expression of the mutant AIRE protein (Figure 1B). This was confirmed by evaluating mRNA expression levels by RT-qPCR (Figure 4). Regarding the effects of these mutations on *Aire* mRNA expression levels, it should be noted that in the murine *Aire* exon 2 KO model, it is still possible to detect mRNA levels comparable to those in the thymi of normal mice (40, 41). Similar to the murine KO model, detection of *Aire* mRNA levels in clone 3.10E6 could be explained by the fact that the mutations occurred in the *Aire* exon 3 CDS region and not at splicing sites or promoter sequences.

To evaluate the effects of the mutations generated by the Crispr-Cas9 system on AIRE protein levels, we used a specific monoclonal antibody that recognizes the region of the protein from amino acid residues 246–545, i.e., the SAND and PHD1–2 domains. WB analysis (Figure 1B) showed that there was a reduction in AIRE protein expression in clone 3.10E6. This may be explained by the fact that one of its alleles encodes a truncated AIRE protein and that the other allele, although it encodes a nonfunctional protein, shows no change in the domains recognized by the primary anti-AIRE antibody used.

Several patients with APECED have mutations in the CARD or NLS domain, and the AIRE protein ceases to be located in the nucleus and is instead deposited in the cytoplasm (47, 49). This observation became of interest for the present work, since the potential consequences of the mutations induced by the Crispr-Cas9 system on AIRE protein function could be due to possible changes in its capacity for intracellular translocation rather than through direct association with DNA or other proteins.

AIRE is a protein that exerts its function in the nucleus, and its nuclear localization occurs due to its NLS domain (45). Mutations generated by the Crispr-Cas9 system occurred within the NLS coding region, which most likely caused perturbations in the nuclear AIRE translocation. This was confirmed through immunofluorescence analysis (Figure 2). Clone 3.10E6, although showing expression of the mutant AIRE protein, does not show localization to the nucleus of mTEC cells. Accordingly, clone 3.10E6 can be considered a KO since the mutant AIRE protein from allele 1, although expressed, had its function altered and its translocation from the cytoplasm to the nucleus impaired. The absence of labeling in the cytoplasm is likely due to insufficient expression of the AIRE protein. Mutations of this type have been described previously in patients with APECED and are, respectively, associated with different symptoms of the disease (39).

Disturbance of *Aire* expression both *in vitro* and *in vivo* leads to changes in regulation of the expression of a large set of genes that are associated with NS, autoantigen processing and other biological processes, and autoantigens themselves (20, 40, 41, 50). In addition, genes encoding adhesion molecules are also perturbed by the reduction of *Aire* expression (20). This led us to evaluate whether *Aire* mutations could affect cell adhesion. Accordingly, we determined whether the mutations induced by Crispr-Cas9 could disrupt the adhesive capacity of mTEC cells with thymocytes, which was demonstrated by means of adhesion assays (Figure 3).

There have been no investigations to date of the link between *Aire*, cell adhesion, and transcriptome profiles of mTECs. However, we find such a relationship plausible since a group of thymocytes expressing RANKL can interact with mTEC cells, induce *Aire* expression (51, 52) and consequently modulate cell adhesion genes (Figure 4) and the transcriptomes of these cells (Figure 6). Since we observed that mTEC-thymocyte adhesion stimulates the expression of *Aire* in mTECs, and considering that *Aire* controls more than 3,300 genes in these cells (11), we next evaluated their transcriptomes. This question was addressed by sequencing the transcriptomes (RNA-Seq) of WT mTECs and 3.10E6 clones from both groups, both before and after thymocyte adhesion.

The mTEC-thymocyte adhesion itself functions as a stimulus for the transcription of mRNAs that encode molecules such as the transcription factor TCF7, a protein that was initially identified in thymocytes and participates in their differentiation in the thymus (53, 54). The *Tcf7* gene has only recently been associated with mTEC cells. It has been shown that this protein is part of an essential transcriptional complex along with other transcription factors, such as *Irf4, Irf8, Tbx21*, and *Ctcfl*, that regulates the expression of *Aire* in these cells (55). We also showed that mTEC-thymocyte adhesion causes increased expression of mRNA transcripts encoding NFKB alpha inhibitor (*Nfkbia*), which is the main inhibitor of the *Nfkb* pathway.

The expression of *Aire* in mTECs is activated by the RANK–RANKL pathway (51, 52) and is also dependent on the binding of NFKB to an enhancer located in a non-coding region between the *Aire* and *Dnmt3L* genes (56). Defects in the Nfkb-mediated signaling pathway also lead to a decrease in *Aire* expression in mTECs (57).

We asked whether the mTEC-thymocyte adhesion that activates the expression of *Tcf7* would activate the *Nfkb* pathway and consequently *Aire* (Figure 4). The increase in *Aire* mRNA expression by mTEC-thymocyte adhesion was found to intensify the AIRE protein localization to the nucleus of WT mTEC 3.10 cells but, as expected, not in clone 3.10E6 cells (Figure 5).

In fact, the transcriptomes of WT mTECs differ from those of clone 3.10E6 cells. This finding has a significant physiological correlation, as the transcriptomes of mTEC^hi^ cells are different from mTEC^lo^ cells, which have low *Aire* expression (11).

The mTEC 3.10 cells are characterized by a CD80^lo^ or MHC-II^lo^ phenotype (20) that, under proper stimulation, initiates *Aire* expression. With altered expression of *Aire*, either by means of anti-*Aire* siRNA (20) or by means of Crispr-Cas9 KO (this study), these cells exhibit marked dysregulation of genes involved with NS. Among the set of mRNAs with dysregulated expression, we have identified several that control one or more of the following processes: cell adhesion, migration, positive regulation of gene expression, signal transduction, or positive regulation of RNA Pol II function.

We have found that several important mRNAs associated with cell adhesion, such as those that encode proteins belonging to the claudins, integrins, selectins, or extracellular matrix protein families, were DE in clone 3.10E6 cells (Figures 4A,B). For example, the mRNAs that encode the *Timp3, Cd24a*, or *Fabp5* proteins, which are important for the occurrence of mTEC-thymocyte interactions, were repressed in clone 3.10E6 cells. Conversely, *Tnc* and *Lgals3bp* mRNAs were induced in this clone. This set of results suggests that thymocyte adhesion and *Aire*, although functioning in different ways, have synergistic activity. Cell adhesion stimulates transcriptional expression in mTEC cells, including of Aire itself, which in turn regulates a downstream cascade of mRNAs that encode cell adhesion molecules.

Evidence suggests that *Aire* plays a role in the processing of mRNAs and therefore contributes to the expression of tissue-specific isoforms in mTECs (17–19). We quantified these isoforms and verified that their abundance was different, but not significantly so, between each of the situations studied. In addition, mTEC-thymocyte adhesion does not appear to affect the abundance of transcripts (Figure 6C). These results corroborate those of St-Pierre et al. (11), who found no differences in the complexity of splicing between mTEC^hi^, mTEC^lo^, and cTEC populations, and Danan-Gotthold et al. (58), who observed equivalent representation of isoforms when comparing WT vs Aire KO mTECs for most tissue-specific transcripts.

However, when analyzing these results in a comparative way, we observed the occurrence of specific transcripts for each of the experimental groups studied. This led us to investigate the influence of *Aire* or mTEC-thymocyte adhesion on isoform modulation. When comparing WT mTEC 3.10 cells with clone 3.10E6 cells (both before and after thymocyte adhesion), it was evident that *Aire* modulates mRNA isoforms that encode cell adhesion molecules.

The occurrence of isoforms in mTECs has an important immunological consequence. This increases the variability of PTAs and consequently increases the chance of negatively selecting autoreactive thymocyte clones in the thymus (17–19, 58). In this work, we have shown that Aire can control the relative abundance of isoforms of mRNAs that encode adhesion molecules. The immunological consequences of this might be associated with the maintenance of different mTEC clones, each exhibiting a different degree of thymocyte adhesion.

As discussed in a previous study from our group (13), healthy individuals also maintain self-reactive T cell clones, and the development of central tolerance is a particularly delicate process during which NS must be sufficiently stringent to avoid the escape of self-reactive T cell clones into the periphery (7).

The results from this work increase our knowledge of the biological function of *Aire* at two levels: (1) the existence of synergy between *Aire* and thymocyte adhesion on the transcriptomes of mTECs and (2) the role played by *Aire* in modulating the expression of mRNAs and their isoforms that encode cell adhesion molecules.

This opens new perspectives for further studies concerning the molecular mechanisms controlling gene expression in mTEC cells upon interaction with thymocytes, as well as the fine-tuning of NS. Finally, considering that mTEC cells are an important target in donor T cell alloimmunity, there is significant implication of these results for further studies of transcriptional expression in the thymus during onset of graft-vs-host-disease (59–61).

## Ethics Statement

Local Animal Ethical Committee of the Ribeirão Preto Medical School, University of São Paulo, Ribeirão Preto Campus, Brazil (permit Number 006/2016-1).

## Author Contributions

CS-H: conceived and performed all the experiments, transfected mTEC cells with Crispr-Cas9 vector, analyzed the mutant mTEC clone, collected, analyzed, and interpreted all data. AA: extracted and analyzed the total RNA samples, analyzed and interpreted the mTEC transcriptome by bioinformatics. RF: transfected mTEC cells with Crispr-Cas9 vector. LC-S: performed the cell adhesion assay. NP and GL: prepared the cell lysate and performed the WB experiments. KB-P: performed the immunofluorescence assays. SG: analyzed and interpreted the mTEC transcriptome by bioinformatics. GP: conceived the study, designed the experiments, decided the Crispr-Cas9 system, interpreted and organized all data, interpreted transcriptome data, and wrote the manuscript.

## Conflict of Interest Statement

The authors declare that the research was conducted in the absence of any commercial or financial relationships that could be construed as a potential conflict of interest.

## Footnotes

^1^https://www.beckman.com/coulter-flow-cytometers/software (Accessed: July 30, 2017).

^2^http://frodo.wi.mit.edu/primer3 (Accessed: July 30, 2017).

^3^http://crispid.gbiomed.kuleuven.be (Accessed: July 30, 2017).

^4^https://www.ncbi.nlm.nih.gov/nuccore/ (Accessed: July 30, 2017).

^5^http://www.uniprot.org/uniprot/ (Accessed: July 30, 2017).

^6^http://web.expasy.org/translate/ (Accessed: July 30, 2017).

^7^http://provean.jcvi.org/index.php (Accessed: July 30, 2017).

^8^http://www.ebi.ac.uk/Tools/msa/clustalo/ (Accessed: July 30, 2017).

^9^http://www.ncbi.nlm.nih.gov/nuccore?itool=toolbar (Accessed: July 30, 2017).

^10^https://www.ibm.com/analytics/data-science/predictive-analytics/spss-statistical-software (Accessed: July 30, 2017).

^11^https://www.bioinformatics.babraham.ac.uk/projects/fastqc/ (Accessed: July 30, 2017).

^12^https://github.com/alexdobin/STAR (Accessed: July 30, 2017).

^13^http://htseq.readthedocs.io (Accessed: July 30, 2017).

^14^https://bioconductor.org/packages/release/bioc/html/DESEq2.html (Accessed: July 30, 2017).

^15^https://www.r-project.org (Accessed: July 30, 2017).

^16^https://deweylab.github.io/RSEM/ (Accessed: July 30, 2017).

^17^http://bioconductor.org/packages/release/bioc/html/EBSeq.html (Accessed: July 30, 2017).

^18^https://www.ncbi.nlm.nih.gov/geo/ (Accessed: July 30, 2017).

^19^https://david.ncifcrf.gov/ (Accessed: July 30, 2017).

## Supplementary Material

Figure S1 Flow cytometry for sorting separation of green-fluorescent-positive (GFP+) mTEC 3.10 cells transfected (or not) with the CRISPR-Cas9 vector.

Figure S2 T7 endonuclease assay. The PCR product of *Aire* gene exon 3 from two wild-type mTEC 3.10 cell samples (mTEC CT1 and mTEC CT2) were used as controls whose 415 bp PCR amplicon was not digested by the T7 enzyme. The PCR amplicon from two mutant clones (mTEC 3.10E6 and mTEC 3.10G10) were digested by the T7 enzyme resulting, as expected, in two DNA fragments with approximately 200 bp. The mTEC 3.10G10 mutant clone was not included in this study. Agilent Bioanalyzer model 2100 microfluidic electrophoresis.

Figure S3 Electropherograms obtained from the Sanger sequencing of the *Aire* exon 3, partial sequence of the 415 bp PCR amplicon. Amplicons of mTEC CT1 and mTEC CT2 cell samples are *Aire* wild-type for both alleles, whereas mTEC 3.10E6 is a compound heterozygous (red rectangle). Position of the PAM sequence is indicated.

Figure S4 Euclidean distances comparing the global differences of the transcriptome as evaluated by RNA-Seq of wild-type mTEC 3.10 (before and after thymocyte adhesion) vs mTEC 3.10E6 mutant clone (before and after thymocyte adhesion).

Figure S5 Image of full Western blot (WB) membrane of SDS-PAGE wild-type mTEC 3.10 and mTEC 3.10E6 mutant clone cell lysates for detection of *Mus musculus* AIRE protein. WB membrane was probed with an antibody against AIRE protein (upper panel), washed and then probed with an antibody against GAPDH that was used as an internal load control.

Table S1 Molecular characterization of the Aire exon 3, mTEC 3.10E6 mutant clone through Sanger DNA sequencing and Provean protein sequence analysis. GenBank NCBI accession numbers: Aire mutant allele 1 (MG493266), Aire mutant allele 2 (MG493265).
