# Supplementary figures and images for: Aire Disruption Influences the Medullary Thymic Epithelial Cell Transcriptome and Interaction With Thymocytes
Source: Front Immunol. 2018 May 7;9:964. doi: 10.3389/fimmu.2018.00964 (PMC5949327; doi:10.3389/fimmu.2018.00964)

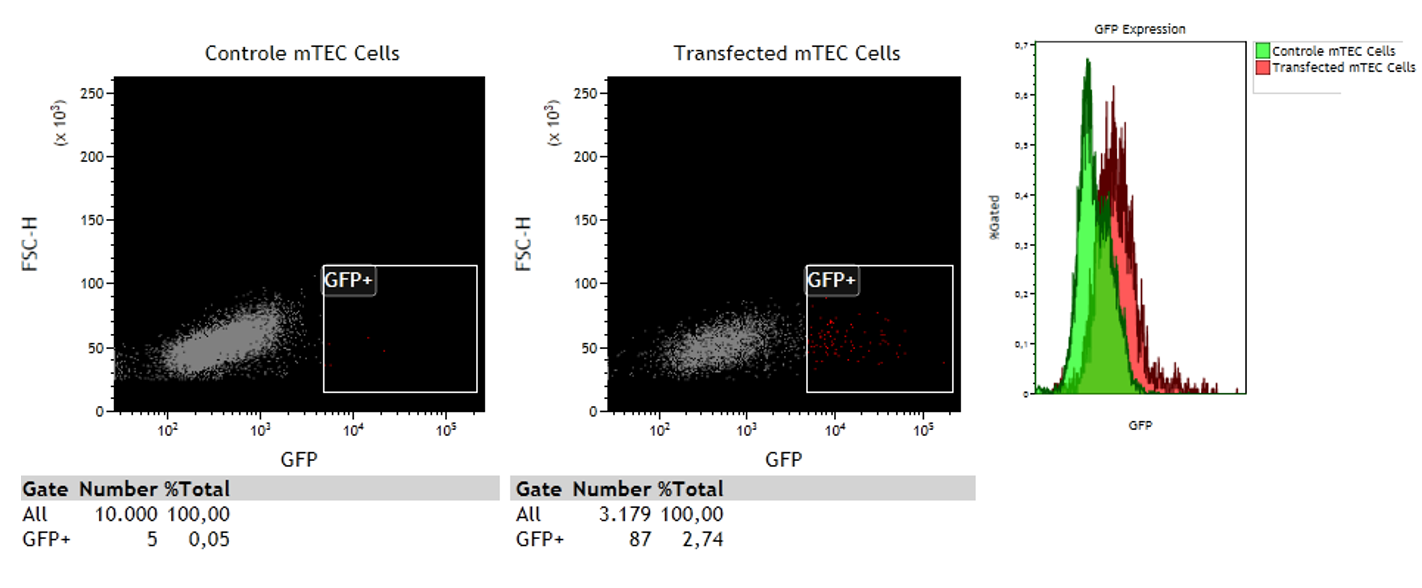

Supplement: Figure S1 — Flow cytometry for sorting separation of green-fluorescent-positive (GFP+) mTEC 3.10 cells transfected (or not) with the CRISPR-Cas9 vector. [file Image_1.TIF]

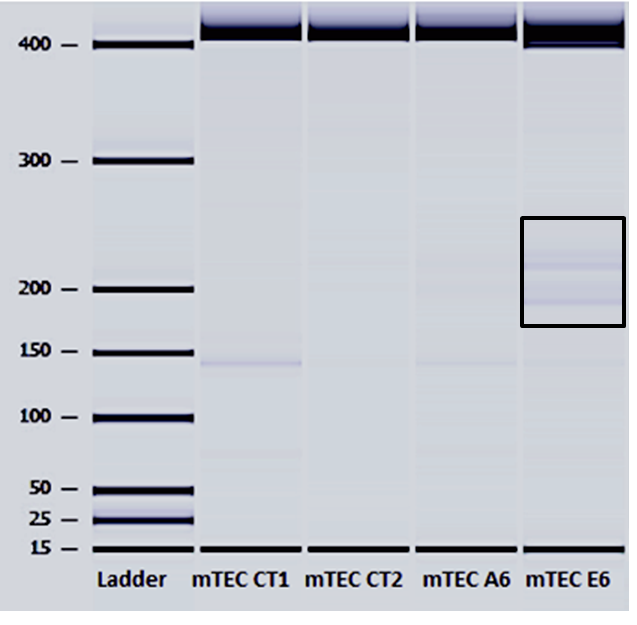

Supplement: Figure S2 — T7 endonuclease assay. The PCR product of Aire gene exon 3 from two wild-type mTEC 3.10 cell samples (mTEC CT1 and mTEC CT2) were used as controls whose 415 bp PCR amplicon was not digested by the T7 enzyme. The PCR amplicon from two mutant clones (mTEC 3.10E6 and mTEC 3.10G10) were digested by the T7 enzyme resulting, as expected, in two DNA fragments with approximately 200 bp. The mTEC 3.10G10 mutant clone was not included in this study. Agilent Bioanalyzer model 2100 microfluidic electrophoresis. [file Image_2.TIF]

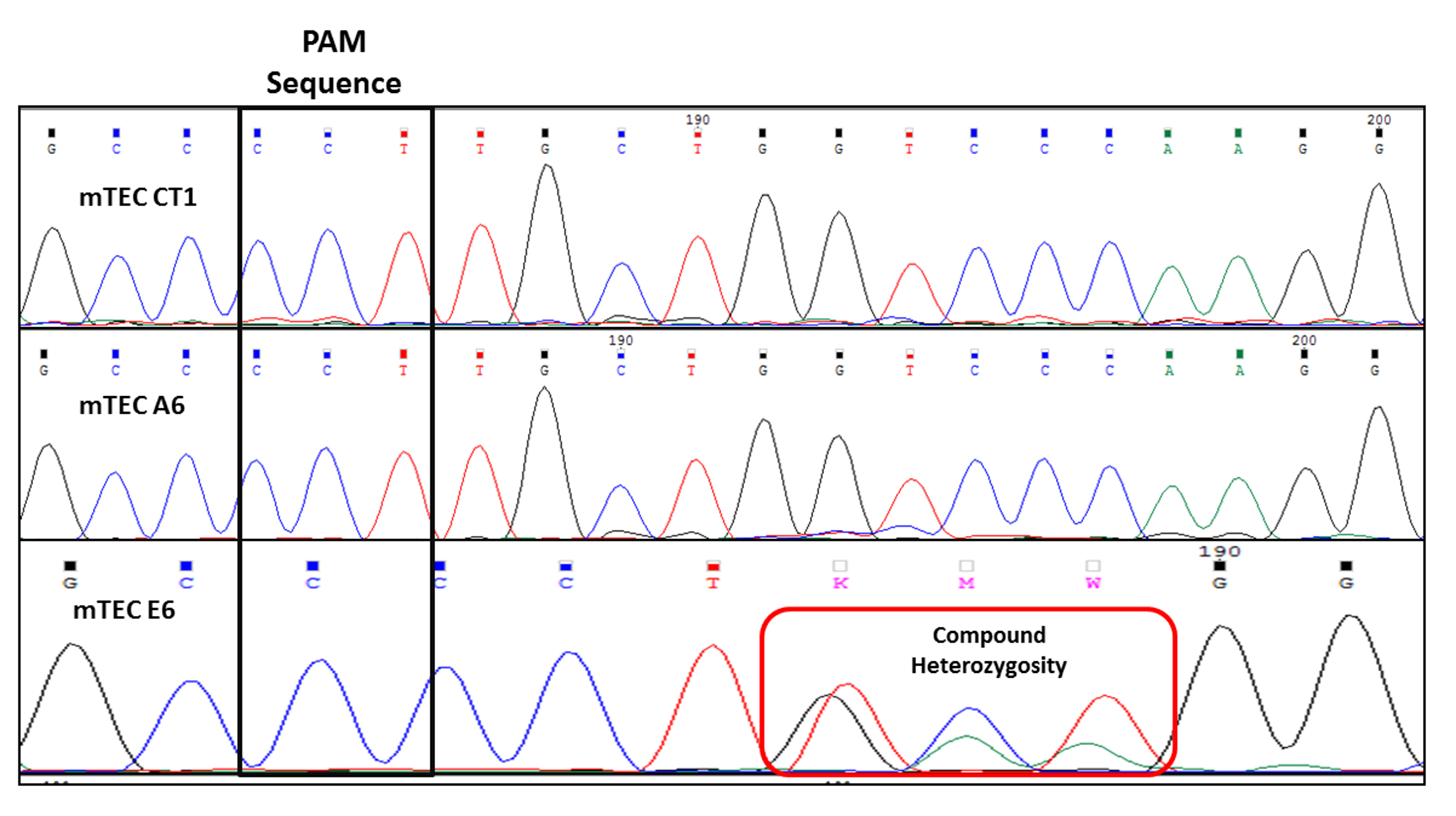

Supplement: Figure S3 — Electropherograms obtained from the Sanger sequencing of the Aire exon 3, partial sequence of the 415 bp PCR amplicon. Amplicons of mTEC CT1 and mTEC CT2 cell samples are Aire wild-type for both alleles, whereas mTEC 3.10E6 is a compound heterozygous (red rectangle). Position of the PAM sequence is indicated. [file Image_3.TIF]

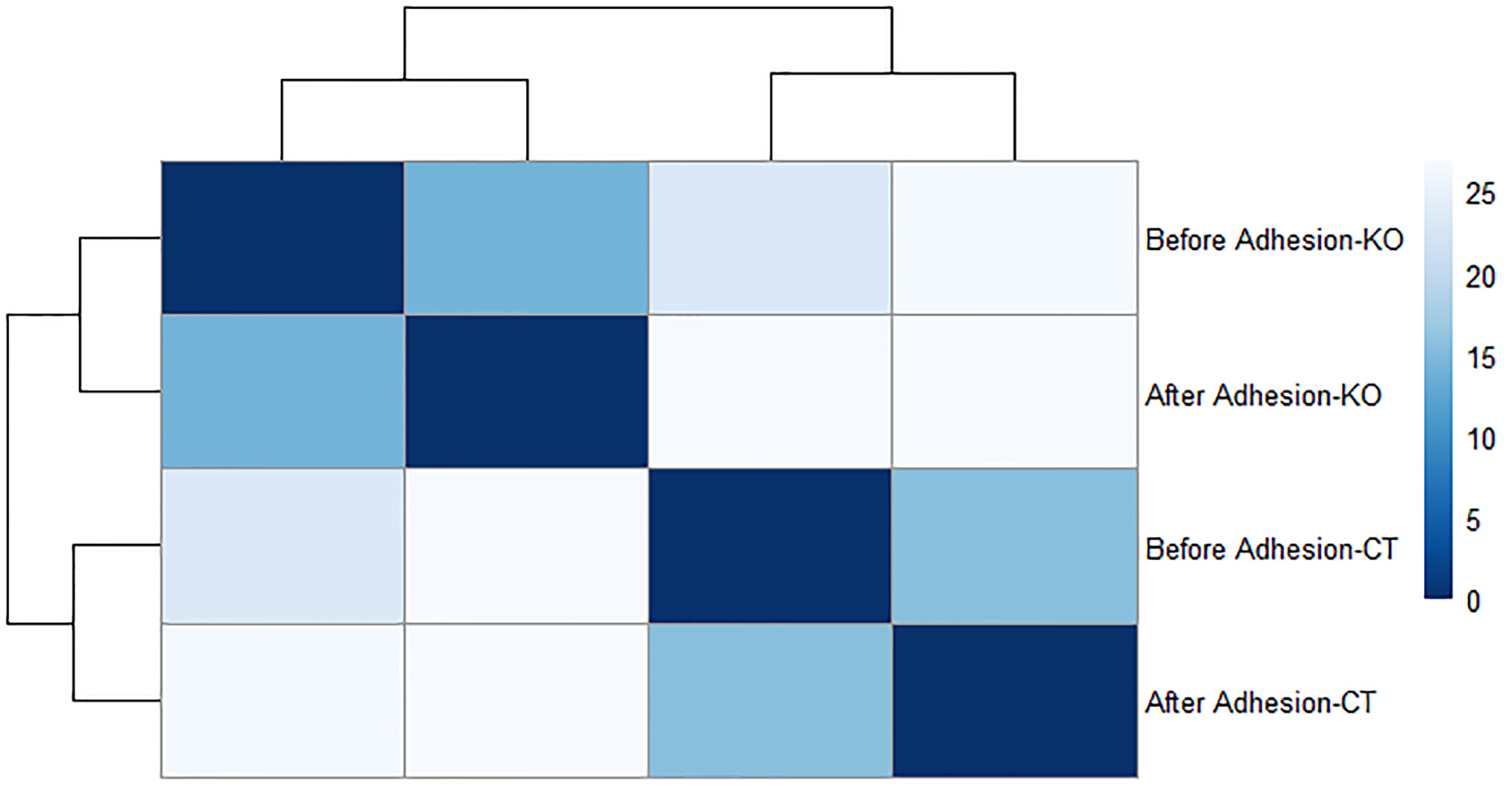

Supplement: Figure S4 — Euclidean distances comparing the global differences of the transcriptome as evaluated by RNA-Seq of wild-type mTEC 3.10 (before and after thymocyte adhesion) vs mTEC 3.10E6 mutant clone (before and after thymocyte adhesion). [file Image_4.TIF]

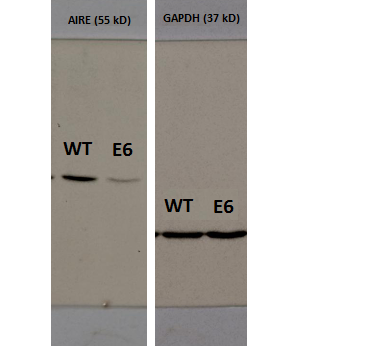

Supplement: Figure S5 — Image of full Western blot (WB) membrane of SDS-PAGE wild-type mTEC 3.10 and mTEC 3.10E6 mutant clone cell lysates for detection of Mus musculus AIRE protein. WB membrane was probed with an antibody against AIRE protein (upper panel), washed and then probed with an antibody against GAPDH that was used as an internal load control. [file Image_5.TIF]
