# Supplementary material for: Aire Disruption Influences the Medullary Thymic Epithelial Cell Transcriptome and Interaction With Thymocytes
Source: Front Immunol. 2018 May 7;9:964. doi: 10.3389/fimmu.2018.00964 (PMC5949327; doi:10.3389/fimmu.2018.00964)
Supplement: Table S1 — Molecular characterization of the Aire exon 3, mTEC 3.10E6 mutant clone through Sanger DNA sequencing and Provean protein sequence analysis. GenBank NCBI accession numbers: Aire mutant allele 1 (MG493266), Aire mutant allele 2 (MG493265). [file Table_1.pdf]

Clone	Allele	Mutation	Position (mRNA)	Type	Frameshift mutation	Effect on Protein	Biological Effect
mTEC E6	Allele 1	T > G	351	Substitution	No	L118L	Silent Mutation
		GCTGGTCCC	352-360	Deletion	No	ΔAGP (119-120)	Deleterious
	Allele 2	G	352	Deletion	Yes (Stop codon TGA in position 474-476)	Truncated Protein 158 aa	Deleterious
